# Supplementary material for: A Combined Human in Silico and CRISPR/Cas9-Mediated in Vivo Zebrafish Based Approach to Provide Phenotypic Data for Supporting Early Target Validation
Source: Front Pharmacol. 2022 Apr 25;13:827686. doi: 10.3389/fphar.2022.827686 (PMC9082939; doi:10.3389/fphar.2022.827686)
Supplement: Supplementary file 1 [file DataSheet1.zip › CRISPR HF (Supp Info including figures and tables).pdf]

SUPPLEMENTARY TABLES

Table S1. List of morphological endpoints scored as a simple yes/no at 2dpf to facilitate selection of treatment groups for more in depth morphological scoring and functional phenotype analysis at 4dpf.

Endpoint	Score criteria	Score	Scored yes/no with notes
**Body shape**	Bent/curved	Yes/No	N/A if not dechorionated*
**Somites**	Poor definition	Yes/No	
**Notochord**	Present / absent	Yes/No	
**Tail**	Bent/curved	Yes/No	N/A if not dechorionated*
**Heart**	No heartbeat	Yes/No	
	Slow heartbeat	Yes/No	
	Swollen pericardial sac	Yes/No	
	Chambers not well defined	Yes/No	
	Misshapen	Yes/No	
**Face**	Optic vesicle small	Yes/No	
	Otic vesicles small	Yes/No	
	Facial oedema	Yes/No	
**Neural**	Irregular shape	Yes/No	Indicate fore, mid or hindbrain
**Jaw/Arches**	Present	Yes/No	
**Body Oedema**	Oedema present	Yes/No	In addition to heart or facial tissue

*Need to be hatched or dechlorinated in advance otherwise a bent body form will persist

Table S2. List of morphological endpoints scored in severity from 1 (severe) to 5 (normal) at 4dpf. Scoring criteria adapted from those of Gustafson *et al*., (2012) and Ball *et al*., (2014).

Endpoint	Score criteria	Score	Notes ~ scored 1 to 5 (normal)*
**Body shape**	Bent/curved	Yes/No	
	Body length	N/A	Standard length in mm
**Somites**	Poor definition	Yes/No	
	Small./ short	Yes/No	
	Missing	Yes/No	
	Cloudy	Yes/No	
**Notochord**	Shortened	Yes/No	
	Folded in Tail	Yes/No	
	No cellular differentiation	Yes/No	
	Wavy/Kinked	Yes/No	
	Poorly defined	Yes/No	
**Tail**	Kinked	Yes/No	
	Bent/Curved	Yes/No	
	Short	Yes/No	
**Fins**	Small	Yes/No	Indicate affected fin(s)
	Irregular Edge	Yes/No	
	Bent	Yes/No	
	Cloudy	Yes/No	
	Eroding	Yes/No	
**Heart**	Slow Heartbeat	Yes/No	
	No Heartbeat	Yes/No	
	Pericardial Sac-Swollen	Yes/No	
	Chambers not well defined	Yes/No	
	Misshapen	Yes/No	
**Face**	Optic Vesicle – Small	Yes/No	
	Optic Vesicle – Misshapen	Yes/No	
	Otic Vesicles – Small	Yes/No	
	Olfactory Region – Reduced	Yes/No	
	Olfactory Region - Not Present	Yes/No	
	Facial Oedema or Hypoplasia	Yes/No	
**Neural Tube**	Irregular Shape	Yes/No	Indicate irregular region(s)
	Reduced/Compressed	Yes/No	
**Arches/Jaw**#	Arches - Not Evident	Yes/No	
	Arches - Irregular Shape	Yes/No	
	Arches – Deficient	Yes/No	
	Jaw - Not Evident	Yes/No	
	Jaw - Deficient	Yes/No	Indicate irregular region(s)
	Jaw - Enlarged/ excessive	Yes/No	
	Jaw - Irregular Shape	Yes/No	
**Liver**	Enlarged	Yes/No	
	Not evident	Yes/No	
**Yolk ball**	Remnant excessive	Yes/No	

#Scoring of the jaw is variable at 4dpf and he jaw at 4dpf, will be more variable at that stage.

Table S3. Sequence of fixation and embedding steps applied to 4dpf zebrafish using an automated tissue embedder.

Solution	Duration	Temperature (ºC)	Vacuum
70% isopropanol	60min	30	on
90% isopropanol	60min	30	on
95% isopropanol	60min	30	on
100% isopropanol	60min	30	on
100% isopropanol	60min	30	on
100% isopropanol	60min	30	on
Xylene	60min	30	on
Xylene	60min	30	on
Xylene	60min	30	on
Wax	80min	62	on
Wax	80min	62	on
Wax	80min	62	on

Table S4. Sequence of H&E staining steps applied to 4dpf zebrafish using an automated stainer.

Solution	Duration
Histoclear	5min
Histoclear	5min
100% IMS	2min
90% IMS	2min
80% IMS	2min
Tap water (running)	2min
Harris Haematoxylin non-acidified	15min
Tap water (running)	2min
Acid Alcohol	5sec
Tap water (running)	30sec
Ammoniated alcohol	30sec
Tap water (running)	30sec
Eosin Y Aqueous	15sec
Tap water (running)	30sec
80% IMS	30sec
90% IMS	1min
95% IMS	1min
100% IMS	2min
100% Ethanol	2min
Histoclear	2min
Histoclear	2min

IMS: Industrial Methylated Spirit

Table S5. Summary of literature data on the structure and function of the genes identified from the *in siico* work for zebrafish-based *in vivo* investigation in the current study

OFFICIAL GENE SYMBOL (ALIASES)	GENE NAME(S)	PROTEIN STRUCTURE & FUNCTION(S)	BIOLOGICAL & PHYSIOLOGICAL FUNCTION(S)
API5<br><br>***(AAC-11; FIF; MIG8)***	Apoptosis Inhibitor 5; Anti-apoptosis clone 11, Fibroblast Growth Factor 2-Interacting Factor;<br>Cell migration-inducing gene 8	•Belongs to the Inhibitor of apoptosis (IAP) family characterized originally as physical inhibitors of caspases (Salvesen et al 2002)<br>•May act as a scaffold for multi-protein complexes. Contains a multiple helices, forming HEAT (Huntingtin, elongation factor 3, PR65/A, TOR)-like and ARM (Armadillo)-like repeats that mediate protein–protein interactions (Han et al 2012),<br>•The C-terminal is structurally similar to the core region of the U-box-containing ubiquitin ligase E4 protein Ufd2p (Han et al 2012)	<u>*Apoptosis*</u><br>•Prevents apoptosis after growth factor deprivation (Tewari et al 1997)<br>•Prevents cell death by negatively regulating E2F1 transcription factor-induced apoptosis (Morris et al 2006). Contributes to E2F1 control of the G1/S cell cycle phase transition via facilitating E2F1 recruitment onto its target promoters and thus E2F1 target gene transcription (Garcia-Jove Navarro et al 2013)<br>•Regulates Acinus (ACIN1), a protein involved in chromatin condensation and DNA fragmentation during apoptosis protecting it from caspase 3 cleavage (Rigou et al 2009)<br>•Binds to and inhibits caspase-2, a key regulator of cell death, autophagy, genomic stability and ageing (Imre et al 2017)<br>•Interacts with FGF2 (Van den Berghe et al 2000) and upregulates FGF2 signaling through a FGFR1/PKCdelta/ERK effector pathway triggering degradation of the proapoptotic molecule BIM (Noh et al 2014)<br>•Regulates miR-1 induced apoptosis (Li et al 2015)<br>•Phosphorylated by PIM2 kinase and inhibits apoptosis in hepatocellular carcinoma cells through NF-κB (nuclear factor-κB) (Ren et al 2010)<br>•Downregulated in cardiomyocytes undergoing H_2O_2-induced apoptosis (Wan et al 2016)<br><u>*Tumorigenesis*</u><br>•Upregulated in human carcinomas in vivo (Koci et al 2012)<br>•Inhibition increases anti-cancer drug sensitivity in various cancer cells (Rigou et al 2009)<br>•Increases the metastatic capacity of tumor cells by upregulating MMP levels via activation of the Erk signaling pathway (Song et al 2015)<br>•Acts as an immune escape gene in tumors by rendering them resistant to

			apoptosis triggered by tumor antigen-specific T cells (Noh et al 2014)<br>*Immune response*<br>• Acts as a danger-associated molecular pattern (DAMP) and stimulate the activation of immune response. Mediates TLR4-dependent activation of antigen presenting cells (Kim et al 2017)
HSPB7<br><br>***(CVHSP)***	Heat Shock Protein Family B (Small) Member 7; Cardiovascular Heat Shock Protein	• Member of the small heat shock proteins (HSPB) family of molecular chaperones. HSPBs typically associate early with misfolded proteins (Mymrikov et al 2011)<br>• Prevents aggregation of proteins with expanded polyglutamine (polyQ) stretches (Vos et al 2010)<br>• Binds large sarcomeric proteins Filamin C and Titin in cardiac cells (Mercer et al 2018)	*Cardiac function*<br>• Selectively expressed in cardiac and skeletal muscle<br>• Common genetic variants associate with human HF (Cappola et al 2010)<br>• Modulates actin filament assembly in heart (Wu et al 2017)<br>• Cardiac-specific inducible HSPB7 KO mice display cardiac arrhythmia with abnormal conduction, rapid HF and sudden death accompanied by a downregulation of intercalated disc proteins and enrichment in Filamin C protein aggregates (Liao et al 2017)<br>• Global depletion of Hspb7 in zebrafish disrupts normal cardiac morphogenesis (Rosenfeld et al 2013)<br>• Loss of HSPB7 in zebrafish or human cardiomyocytes leads to enhanced autophagic pathways (Mercer et al 2018)<br>• Dramatically increased in the heart and blood plasma immediately after myocardial infarction (Rüdebusch et al 2017)<br>*Tumorigenesis*<br>• May act as a tumor suppressor in the p53 pathway (Lin et al 2014)
LMO2<br><br>***(TTG2; RBTN2; RHOM2)***	LIM Domain Only 2; Rhombotin-2	• Belongs to the LIM-Only group of LIM domain protein superfamily.<br>• The LIM-fingers are distinct from the zinc-fingers of transcription factors such as *GATA*, as LIM-domain-only proteins do not directly bind to DNA (8)<br>• Mediates transactivation via protein-protein interactions with other transcriptional factors.	*Angiogenesis*<br>• Regulates endothelial proliferation and angiogenesis *in vitro* and is required for angiogenesis and tissue healing *in vivo* (Meng et al 2016)<br>• Promotes endothelial cell migration via modulation of Sphk1 (Sphingosine Kinase). *Lmo2* KD reduced Lmo2-Sphk1 gene interaction, impaired intersegmental vessels formation, and reduced cell migration (Matrone et al 2017)<br>• Angiopoietin-2 and VE-cadherin, both major regulators of angiogenesis, are direct transcriptional targets of LMO2-complexes with bHLH transcription factors TAL1 and/or LYL1 in endothelial cells (Deleuze et al. 2007, 2012)<br>• Regulate VEGF-induced angiogenesis and lymphangiogenesis via NRP2 (Coma et al 2013)

			<u>*Hematopoiesis/Erythropoiesis*</u><br>• Homozygous inactivation in mice led to the complete absence of yolk sac erythropoiesis and early embryonic lethality (Warren et al. 1994)<br>• Acts with TAL1/SCL to regulate erythropoiesis (Valge-Archer et al 1994)<br><u>*Tumorigenesis*</u><br>• Attenuates tumor growth by targeting the Wnt signaling pathway in breast and colorectal cancer (Liu et al 2016)<br>• Regulator of neo-vascularization of tumors (Yamada et al. 2002)<br>• Acts as a proto-oncogene in T cells (Mao et al 1997). Recurrent findings of interstitial deletions and translocations involving *LMO2* in T cell leukaemias.

References from Table S5

API5:

Wan C, Xiang J, Li Y, Guo D. Differential Gene Expression Patterns in Chicken Cardiomyocytes during Hydrogen Peroxide-Induced Apoptosis. Plos one. 2016;11(1):e0147950. DOI: 10.1371/journal.pone.0147950.

Song KH, Kim SH, Noh KH, et al. Apoptosis Inhibitor 5 Increases Metastasis via Erk-mediated MMP expression. BMB Reports. 2015 Jun;48(6):330-335. DOI: 10.5483/bmbrep.2015.48.6.139.

Li D, Liu Y, Li H, et al. MicroRNA-1 promotes apoptosis of hepatocarcinoma cells by targeting apoptosis inhibitor-5 (API-5). FEBS Letters. 2015 Jan;589(1):68-76. DOI: 10.1016/j.febslet.2014.11.025.

Noh KH, Kim SH, Kim JH, et al. API5 confers tumoral immune escape through FGF2-dependent cell survival pathway. Cancer Research. 2014 Jul;74(13):3556-3566. DOI: 10.1158/0008-5472.can-13-3225.

Garcia-Jove Navarro M, Basset C, Arcondéguy T, et al. Api5 contributes to E2F1 control of the G1/S cell cycle phase transition. Plos one. 2013 ;8(8):e71443. DOI: 10.1371/journal.pone.0071443.

Koci L, Chlebova K, Hyzdalova M, et al. Apoptosis inhibitor 5 (API-5; AAC-11; FIF) is upregulated in human carcinomas in vivo. Oncology Letters. 2012 Apr;3(4):913-916. DOI: 10.3892/ol.2012.593.

Han BG, Kim KH, Lee SJ, et al. Helical repeat structure of apoptosis inhibitor 5 reveals protein-protein interaction modules. The Journal of Biological Chemistry. 2012 Mar;287(14):10727-10737. DOI: 10.1074/jbc.m111.317594.

Morris EJ, Michaud WA, Ji JY, et al. Functional identification of Api5 as a suppressor of E2F-dependent apoptosis in vivo. Plos Genetics. 2006 Nov;2(11):e196. DOI: 10.1371/journal.pgen.0020196.

Tewari M, Yu M, Ross B, et al. AAC-11, a novel cDNA that inhibits apoptosis after growth factor withdrawal. Cancer Research. 1997 Sep;57(18):4063-4069.

Ren K, Zhang W, Shi Y, Gong J. Pim-2 activates API-5 to inhibit the apoptosis of hepatocellular carcinoma cells through NF-kappaB pathway. Pathology Oncology Research : POR. 2010 Jun;16(2):229-237. DOI: 10.1007/s12253-009-9215-4.

Salvesen GS, Duckett CS. IAP proteins: blocking the road to death's door. Nature reviews. Molecular Cell Biology. 2002 Jun;3(6):401-410. DOI: 10.1038/nrm830.

Van den Berghe L, Laurell H, Huez I, et al. FIF [fibroblast growth factor-2 (FGF-2)-interacting-factor], a nuclear putatively antiapoptotic factor, interacts specifically with FGF-2. Molecular Endocrinology (Baltimore, Md.). 2000 Nov;14(11):1709-1724. DOI: 10.1210/mend.14.11.0556.
Rigou P, Piddubnyak V, Faye A, et al. The antiapoptotic protein AAC-11 interacts with and regulates Acinus-mediated DNA fragmentation. The EMBO Journal. 2009 Jun;28(11):1576-1588. DOI: 10.1038/emboj.2009.106.
Kim YS, Park HJ, Park JH, et al. A novel function of API5 (apoptosis inhibitor 5), TLR4-dependent activation of antigen presenting cells. Oncoimmunology. 2018 ;7(10):e1472187. DOI: 10.1080/2162402x.2018.1472187.
Imre G, Berthelet J, Heering J, et al. Apoptosis inhibitor 5 is an endogenous inhibitor of caspase-2. EMBO Reports. 2017 May;18(5):733-744. DOI: 10.15252/embr.201643744.
HSPB7:
Liao WC, Juo LY, Shih YL, Chen YH, Yan YT. HSPB7 prevents cardiac conduction system defect through maintaining intercalated disc integrity. PLoS Genet. 2017 Aug 21;13(8):e1006984. doi: 10.1371/journal.pgen.1006984. PMID: 28827800; PMCID: PMC5587339.
Mymrikov EV, Seit-Nebi AS, Gusev NB. Large potentials of small heat shock proteins. Physiol Rev. 2011 Oct;91(4):1123-59. doi: 10.1152/physrev.00023.2010. PMID: 22013208.
Wu T, Mu Y, Bogomolovas J, Fang X, Veevers J, Nowak RB, Pappas CT, Gregorio CC, Evans SM, Fowler VM, Chen J. HSPB7 is indispensable for heart development by modulating actin filament assembly. Proc Natl Acad Sci U S A. 2017 Nov 7;114(45):11956-11961. doi: 10.1073/pnas.1713763114. Epub 2017 Oct 23. PMID: 29078393; PMCID: PMC5692592.
Cappola TP, Li M, He J, et al. Common variants in HSPB7 and FRMD4B associated with advanced heart failure. Circulation. Cardiovascular Genetics. 2010 Apr;3(2):147-154. DOI: 10.1161/circgenetics.109.898395.
Rüdebusch J, Benkner A, Poesch A, et al. Dynamic adaptation of myocardial proteome during heart failure development. Plos one. 2017 ;12(10):e0185915. DOI: 10.1371/journal.pone.0185915.
Vos MJ, Zijlstra MP, Kanon B, van Waarde-Verhagen MA, Brunt ER, Oosterveld-Hut HM, Carra S, Sibon OC, Kampinga HH. HSPB7 is the most potent polyQ aggregation suppressor within the HSPB family of molecular chaperones. Hum Mol Genet. 2010 Dec 1;19(23):4677-93. doi: 10.1093/hmg/ddq398. Epub 2010 Sep 15. PMID: 20843828.
Rosenfeld GE, Mercer EJ, Mason CE, Evans T. Small heat shock proteins Hspb7 and Hspb12 regulate early steps of cardiac morphogenesis. Developmental Biology. 2013 Sep;381(2):389-400. DOI: 10.1016/j.ydbio.2013.06.025.
Mercer EJ, Lin YF, Cohen-Gould L, Evans T. Hspb7 is a cardioprotective chaperone facilitating sarcomeric proteostasis. Developmental Biology. 2018 Mar;435(1):41-55. DOI: 10.1016/j.ydbio.2018.01.005.
Lin J, Deng Z, Tanikawa C, Shuin T, Miki T, Matsuda K, Nakamura Y. Downregulation of the tumor suppressor HSPB7, involved in the p53 pathway, in renal cell carcinoma by hypermethylation. Int J Oncol. 2014 May;44(5):1490-8. doi: 10.3892/ijo.2014.2314. Epub 2014 Feb 27. PMID: 24585183; PMCID: PMC4027944.
LMO2:
Chambers J, Rabbitts TH. LMO2 at 25 years: a paradigm of chromosomal translocation proteins. Open Biol. 2015 Jun;5(6):150062. doi: 10.1098/rsob.150062. PMID: 26108219; PMCID: PMC4632508.
Matrone G, Meng S, Gu Q, et al. Lmo2 (LIM-Domain-Only 2) Modulates Sphk1 (Sphingosine Kinase) and Promotes Endothelial Cell Migration. Arteriosclerosis, Thrombosis, and Vascular Biology. 2017 Oct;37(10):1860-1868. DOI: 10.1161/atvbaha.117.309609.

Deleuze V, El-Hajj R, Chalhoub E, Dohet C, Pinet V, Couttet P, Mathieu D. Angiopoietin-2 is a direct transcriptional target of TAL1, LYL1 and LMO2 in endothelial cells. PLoS One. 2012;7(7):e40484. doi: 10.1371/journal.pone.0040484. Epub 2012 Jul 6. PMID: 22792348; PMCID: PMC3391236.

Deleuze V, Chalhoub E, El-Hajj R, Dohet C, Le Clech M, Couraud PO, Huber P, Mathieu D. TAL-1/SCL and its partners E47 and LMO2 up-regulate VE-cadherin expression in endothelial cells. Mol Cell Biol. 2007 Apr;27(7):2687-97. doi: 10.1128/MCB.00493-06. Epub 2007 Jan 22. PMID: 17242194; PMCID: PMC1899886.

Coma S, Allard-Ratick M, Akino T, van Meeteren LA, Mammoto A, Klagsbrun M. GATA2 and Lmo2 control angiogenesis and lymphangiogenesis via direct transcriptional regulation of neuropilin-2. Angiogenesis. 2013 Oct;16(4):939-52. doi: 10.1007/s10456-013-9370-9. Epub 2013 Jul 28. PMID: 23892628; PMCID: PMC3793839.

Liu Y, Huang D, Wang Z, Wu C, Zhang Z, Wang D, Li Z, Zhu T, Yang S, Sun W. LMO2 attenuates tumor growth by targeting the Wnt signaling pathway in breast and colorectal cancer. Sci Rep. 2016 Oct 25;6:36050. doi: 10.1038/srep36050. PMID: 27779255; PMCID: PMC5078767.

Warren AJ, Colledge WH, Carlton MB, Evans MJ, Smith AJ, Rabbitts TH. The oncogenic cysteine-rich LIM domain protein rbtn2 is essential for erythroid development. Cell. 1994 Jul 15;78(1):45-57. doi: 10.1016/0092-8674(94)90571-1. PMID: 8033210.

Yamada, Y., Pannell, R., Forster, A. et al. The LIM-domain protein Lmo2 is a key regulator of tumour angiogenesis: a new anti-angiogenesis drug target. Oncogene 21, 1309–1315 (2002). https://doi.org/10.1038/sj.onc.1205285

Valge-Archer VE, Osada H, Warren AJ, Forster A, Li J, Baer R, Rabbitts TH. The LIM protein RBTN2 and the basic helix-loop-helix protein TAL1 are present in a complex in erythroid cells. Proc Natl Acad Sci U S A. 1994 Aug 30;91(18):8617-21. doi: 10.1073/pnas.91.18.8617. PMID: 8078932; PMCID: PMC44657.

Meng S, Matrone G, Lv J, Chen K, Wong WT, Cooke JP. LIM Domain Only 2 Regulates Endothelial Proliferation, Angiogenesis, and Tissue Regeneration. J Am Heart Assoc. 2016 Oct 6;5(10):e004117. doi: 10.1161/JAHA.116.004117. PMID: 27792641; PMCID: PMC5121509.

Visvader JE, Mao X, Fujiwara Y, Hahm K, Orkin SH. The LIM-domain binding protein Ldb1 and its partner LMO2 act as negative regulators of erythroid differentiation. Proc Natl Acad Sci U S A. 1997 Dec 9;94(25):13707-12. doi: 10.1073/pnas.94.25.13707. PMID: 9391090; PMCID: PMC28370.

Mao S, Neale GA, Goorha RM. T-cell proto-oncogene rhombotin-2 is a complex transcription regulator containing multiple activation and repression domains. J Biol Chem. 1997 Feb 28;272(9):5594-9. doi: 10.1074/jbc.272.9.5594. PMID: 9038167.

SUPPLEMENTARY FIGURES

A

gata5

gRNA	Target sequence (5'-3')
g#1	GATAACTCTTCGTTCAACCCCGG
g#2	GAGCATGGCTGGTACACGAGTGG
g#3	TCCCGGAATCGTGTGCGTAGGGG

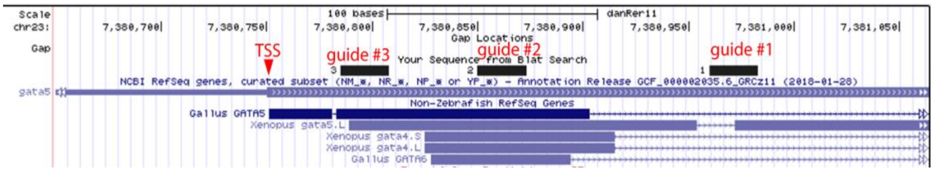

api5

gRNA	Target sequence (5'-3')
g#1	CGCACAGCTCGATCTGTGTGAGG
g#2	CATCCTCGCCGACGCCAAAGAGG
g#3	CGGCGAGGATGCCATAGTTACGG

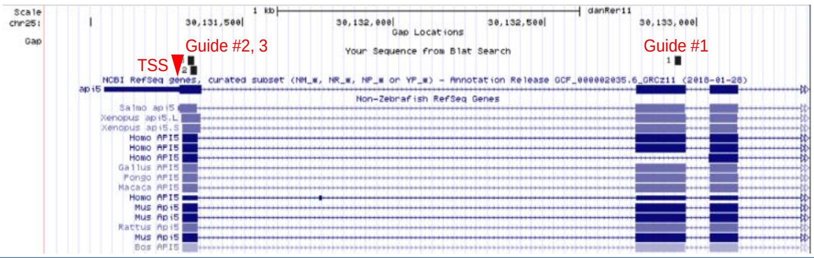

hspb7

gRNA	Target sequence (5'-3')
g#1	CATGTGTCCGAAGGATGCTTTGG
g#2	CCATACATGGAGAAGAGCCGAGG
g#3	CAATTCTTCTGCCTATCGATCGG

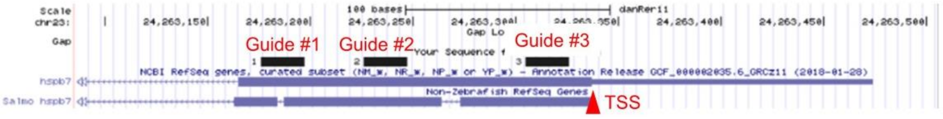

lmo2

gRNA	Target sequence (5'-3')
g#1	CGATGGCCTTCAGAAAGAAGCGG
g#2	GGCGGGTGTCAGCAGAGCATCGG
g#3	TGCGTCCCACCTCCCCTAAGCGG

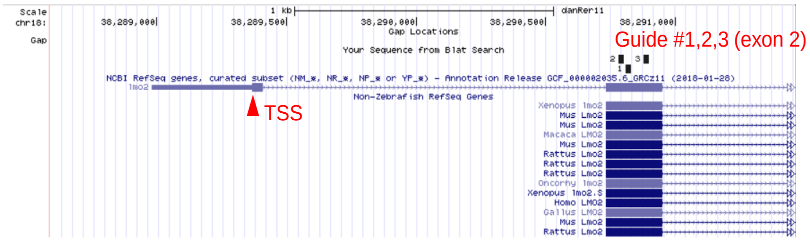

B

gRNA	Target sequence (5'-3')	Efficiency score[a]	On-target score[b]	Off-target score[b]	Number of off-targets for mismatches (MMs) 0 MM	1 MM	2 MMs	3 MMs
gata5-g#1	GATAACTCTTCGTTCAACCCCGG	70	62	94	0	0	0	0
gata5-g#2	GAGCATGGCTGGTACACGAGTGG	69	52	95	0	0	0	0
gata5-g#3	TCCCGGAATCGTGTGCGTAGGGG	62	23	99	0	0	0	0
api5-g#1	CGCACAGCTCGATCTGTGTGAGG	62	8	87	0	0	0	0
api5-g#2	CATCCTCGCCGACGCCAAAGAGG	58	45	96	0	0	0	0
api5-g#3	CGGCGAGGATGCCATAGTTACGG	49	34	96	0	0	0	0
hspb7-g#1	CATGTGTCCGAAGGATGCTTTGG	37	40	93	0	0	0	0
hspb7-g#2	CCATACATGGAGAAGAGCCGAGG	66	68	91	0	0	0	1
hspb7-g#3	CAATTCTTCTGCCTATCGATCGG	62	67	87	0	0	0	1
lmo2-g#1	CGATGGCCTTCAGAAAGAAGCGG	63	41	71	0	0	0	0
lmo2-g#2	GGCGGGTGTCAGCAGAGCATCGG	55	19	81	0	0	0	0
lmo2-g#3	TGCGTCCCACCTCCCCTAAGCGG	62	63	96	0	0	0	0

PAM sequences (NGG) are underlined.

[a] Scores were obtained using CHOPCHOP (https://chopchop.cbu.uib.no/). Setting: Doench *et al*. (2016) *Nature Biotechnology*, 34, p184–191

[b] Scores were obtained using IDT CRISPR-Cas9 gRNA checker (https://www.idtdna.com/Cas9Checker)

Fig. S1. gRNA design for each of the 4 candidate genes. Panel A) For each gene in turn, the left hand table shows the sequence of each designed gRNA (g#1,2,3 encompassed all three gRNAs in one injection), and the right hand image shows the position of each of these gRNA sequences within the coding exons of each gene. The translation start site is indicated by TSS and the adjoining arrow, and other species orthologues are shown for comparison. Original images obtained using the UCS genome browser (https://genome.ucsc.edu). **Panel B)** Table showing the CHOPCHOP scores for efficiency, target specificity, and mismatches detected for each gRNA designed.

A

	Malformations at 4 days post fertilisation (4dpf)																			
	n-number (scored)	Larvae lethality	Larvae unhatched	Larvae abnormal	Body shape	Somites	Notochord	Tail	Fins	Heart	Face	Neural tube	Arches/Jaws	Group mean length (mm)	Swim bladder not inflated	Swim bladder not evident	Yolk ball - remnant excessive	Liver - not evident	Liver -enlarged	Abdomen distended/yolk sac edema
**Un-injected**	10	0%	0%	40%	0%	0%	0%	0%	0%	40%	0%	0%	0%	3.65	0%	0%	0%	0%	0%	0%
**Cas 9 only**	20	0%	0%	20%	0%	0%	0%	0%	0%	20%	0%	0%	0%	3.58	50%	0%	0%	0%	0%	0%
***gata5* g#1,2,3 only**	10	0%	0%	10%	0%	0%	0%	0%	0%	10%	0%	0%	0%	3.75	0%	0%	0%	0%	0%	0%
***gata5* g#1 + Cas9**	10	0%	0%	100%	10%	50%	0%	0%	40%	100%	60%	60%	80%	3.31	100%	60%	0%	60%	0%	50%
***gata5* g#1,2,3 + Cas9**	20	0%	0%	100%	0%	80%	20%	0%	90%	100%	85%	90%	95%	3.15	100%	85%	0%	25%	0%	75%

	% Cardiac specific abnormalities						
	Slow Heartbeat	No Heartbeat	Pericardial Sac-Swollen	Chambers not well defined	Misshapen	Small	Enlarged
**Un-injected**	0%	0%	40%	0%	0%	0%	0%
**Cas 9 only**	0%	0%	20%	0%	0%	0%	0%
***gata5* g#1,2,3 only**	0%	0%	10%	0%	0%	0%	0%
***gata5* g#1 + Cas9**	0%	0%	100%	60%	60%	60%	0%
***gata5* g#1,2,3 + Cas9**	0%	5%	100%	40%	85%	85%	0%

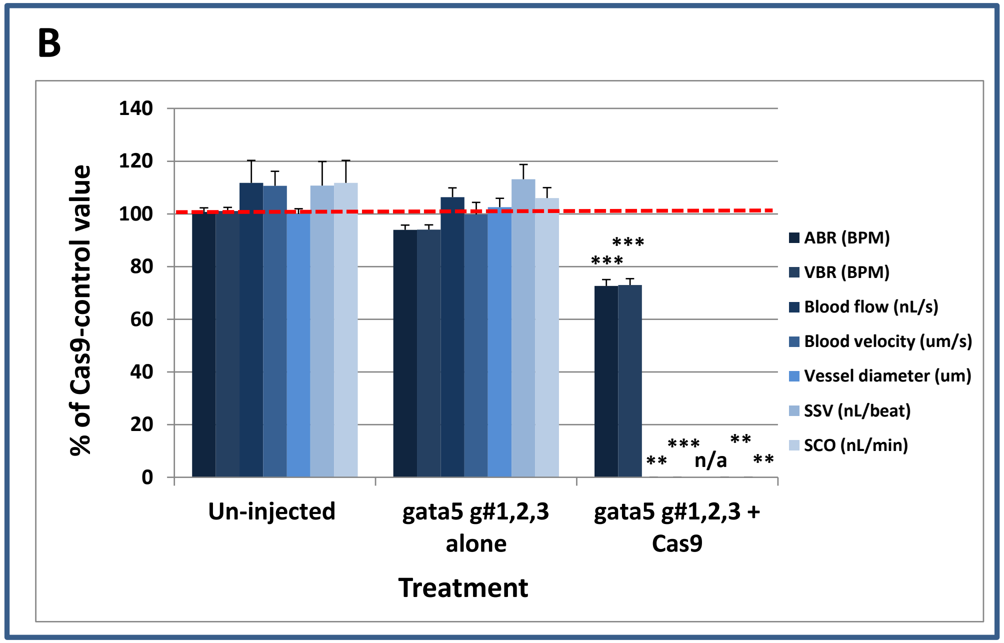

Fig. S2. Comparison of the data from various control treated 4dpf larvae. The control groups assessed were un-injected, Cas9 only injected, *gata5* g#1,2,3 only injected and *gata5* g#1,2,3 + Cas9 injected (positive control). **Panel A)** Summary of the morphological data obtained from these animals including general and heart specific endpoints. Data are shown as the % incidence of abnormalities under each category, with shades from white (0%) through to red (100%) providing an indication of the proportion of animals exhibiting a malformation within that category. Note the low incidence of abnormalities in each treatment except for the positive control group. **Panel B)** Summary of the cardiovascular function data obtained from these animals. Data are shown as the mean % change versus the Cas9-only injected group (100% indicated by the red dashed line), ± SEM, n=9-10. **signifies a statistically significant difference versus the Cas9 control at p<0.01, and *** at p<0.001 (1-way ANOVA and Tukey's HSD tests or Kruskal-Wallis and Dunn's tests). Vessel diameter measurements not possible where no blood flow (n/a).

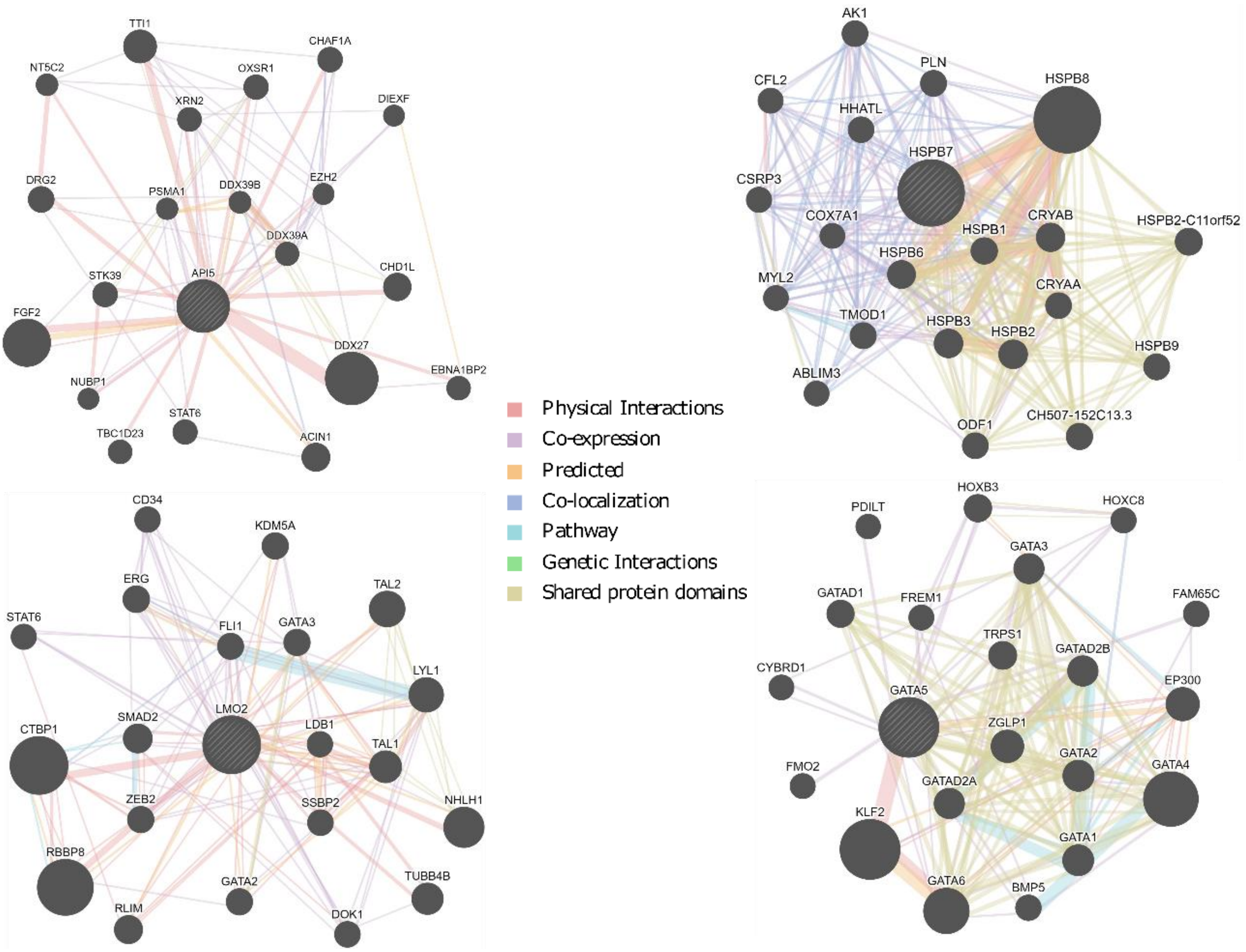

Fig. S3. GeneMANIA network analysis using *API5*, *HSPB7*, *LMO2* and *GATA5* as input genes. GeneMANIA uses very large set of functional association data including protein and genetic interactions, pathways, co-expression, co-localization and protein domain similarity (Warde-Farley *et al*., 2010. *Nucleic Acids Res*, 38, W214-20). Colour of network edges indicate evidence types.

i) GATA5 - Enhanced in testis, bladder, stomach and female reproductive organs

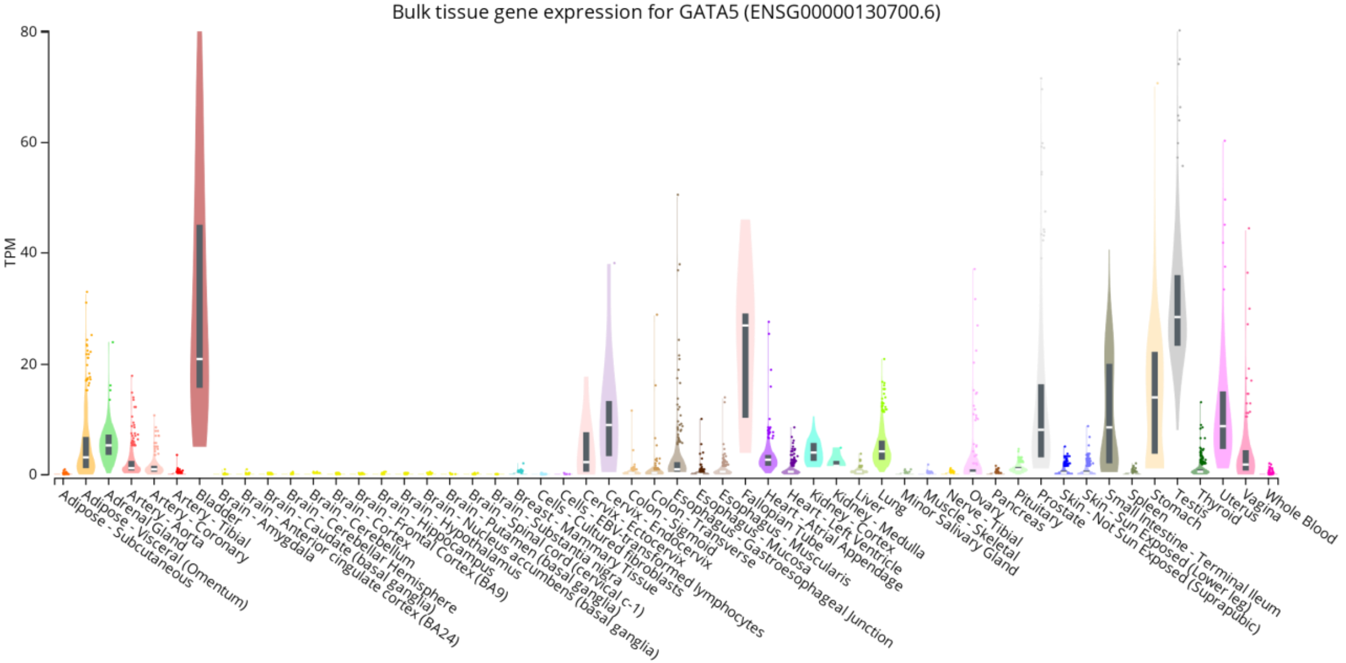

iii) GATA5 – Generally weak expression. Highest in Cardiomyocytes (atrial)

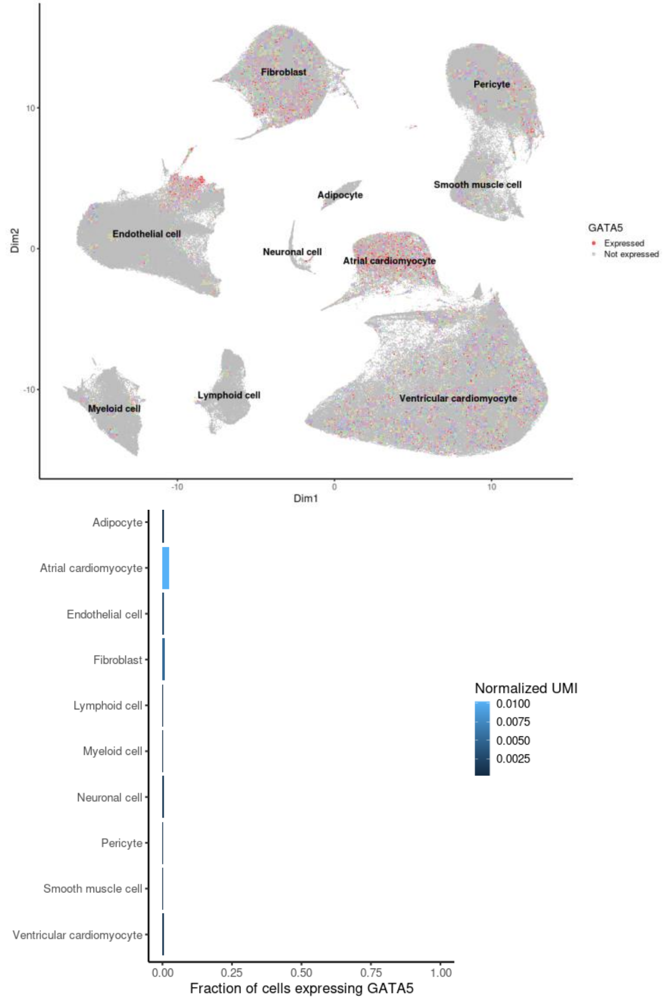

ii) GATA5 - Enhanced in Hepatic stellate (Ito) cells and Cardiomyocytes

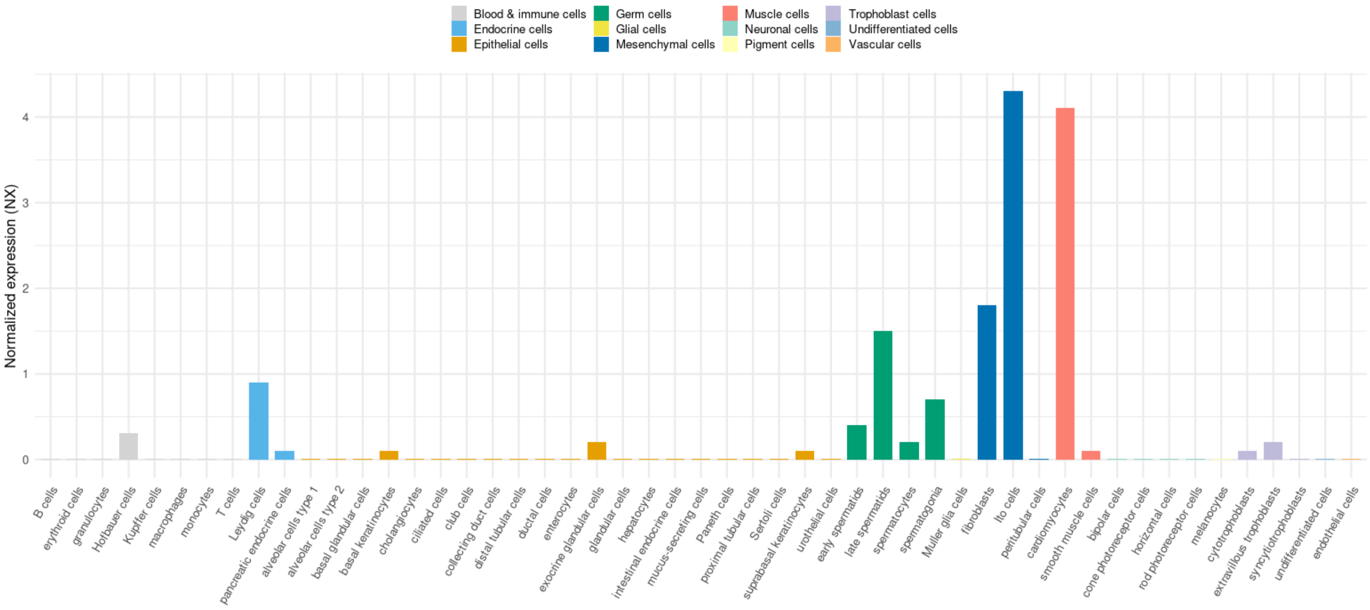

Fig. S4A. Transcriptomics data analysis on *GATA5*. Data shown are the tissue and single cell type mRNA expression levels based on RNA sequencing data sets from the **i)** GTExPortal (https://gtexportal.org/home/), **ii)** the Human Protein Atlas (https://www.proteinatlas.org/) and **iii)** the Heart Cell Atlas (https://www.heartcellatlas.org/).

i) API5 – Ubiquitous. Low tissue specificity

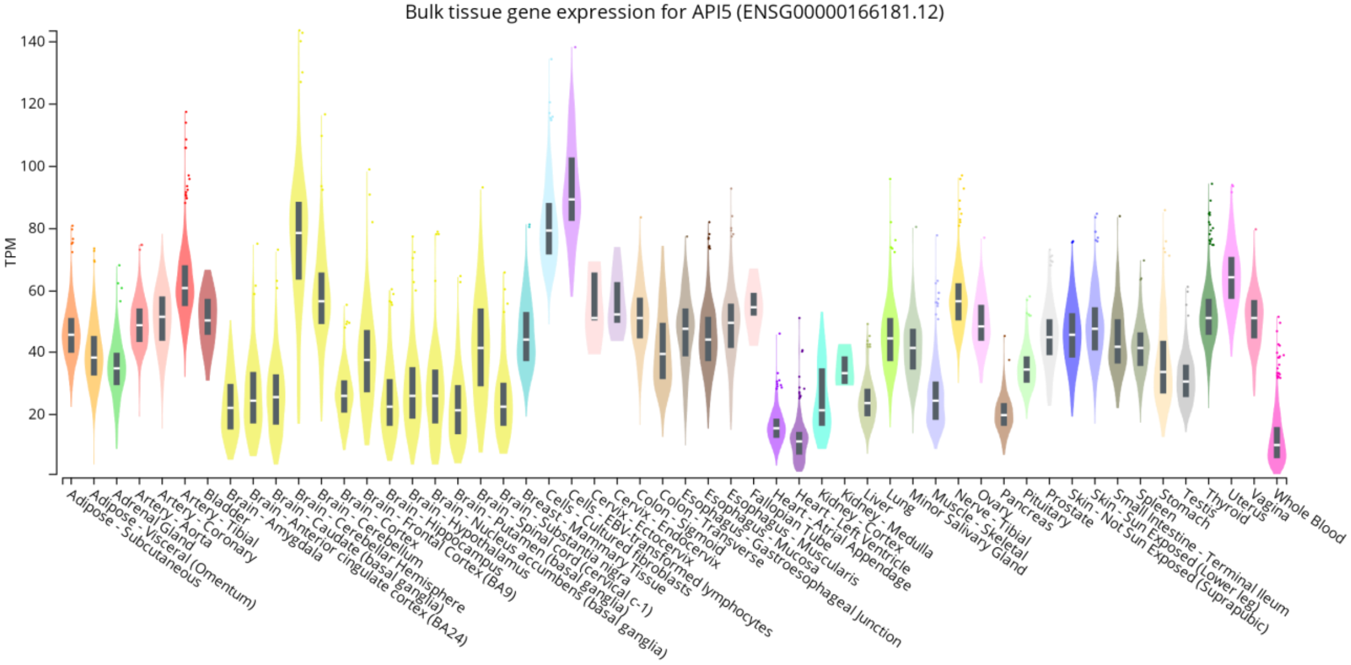

ii) API5 – Ubiquitous. Low cell type specificity

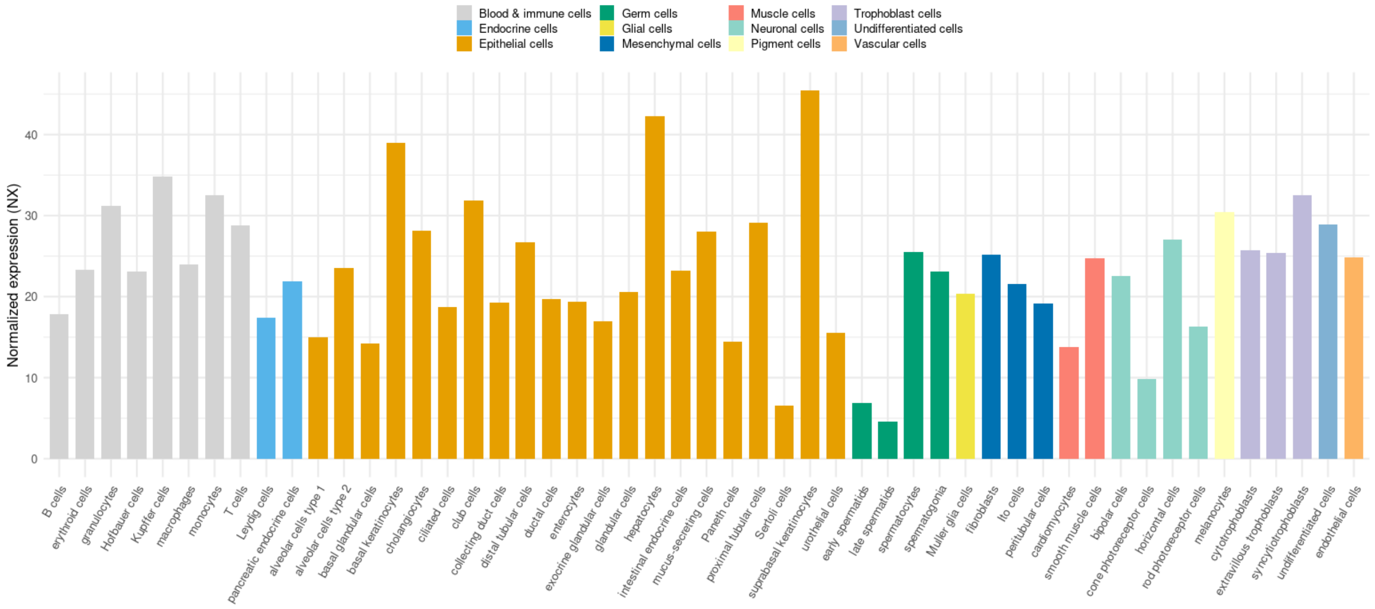

iii) API5 – Ubiquitous. Low cell type specificity

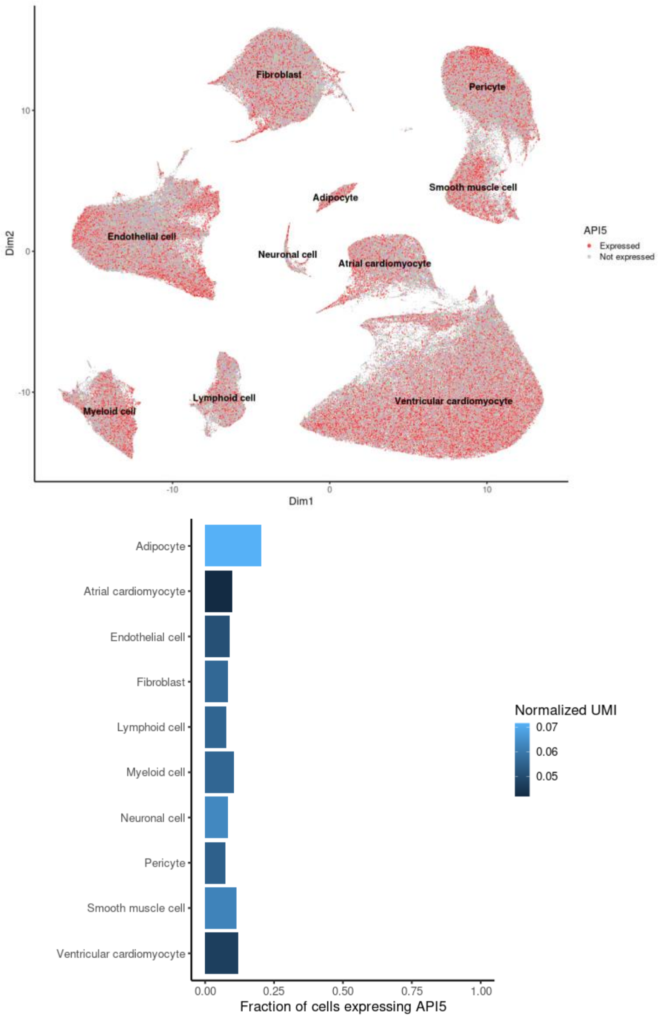

Fig. S4B. Transcriptomics data analysis on *API5*. Data shown are the tissue and single cell type mRNA expression levels based on RNA sequencing data sets from the **i)** GTExPortal (https://gtexportal.org/home/), **ii)** the Human Protein Atlas (https://www.proteinatlas.org/) and **iii)** the Heart Cell Atlas (https://www.heartcellatlas.org/).

i) HSPB7 - Enriched in enriched heart muscle and skeletal muscle

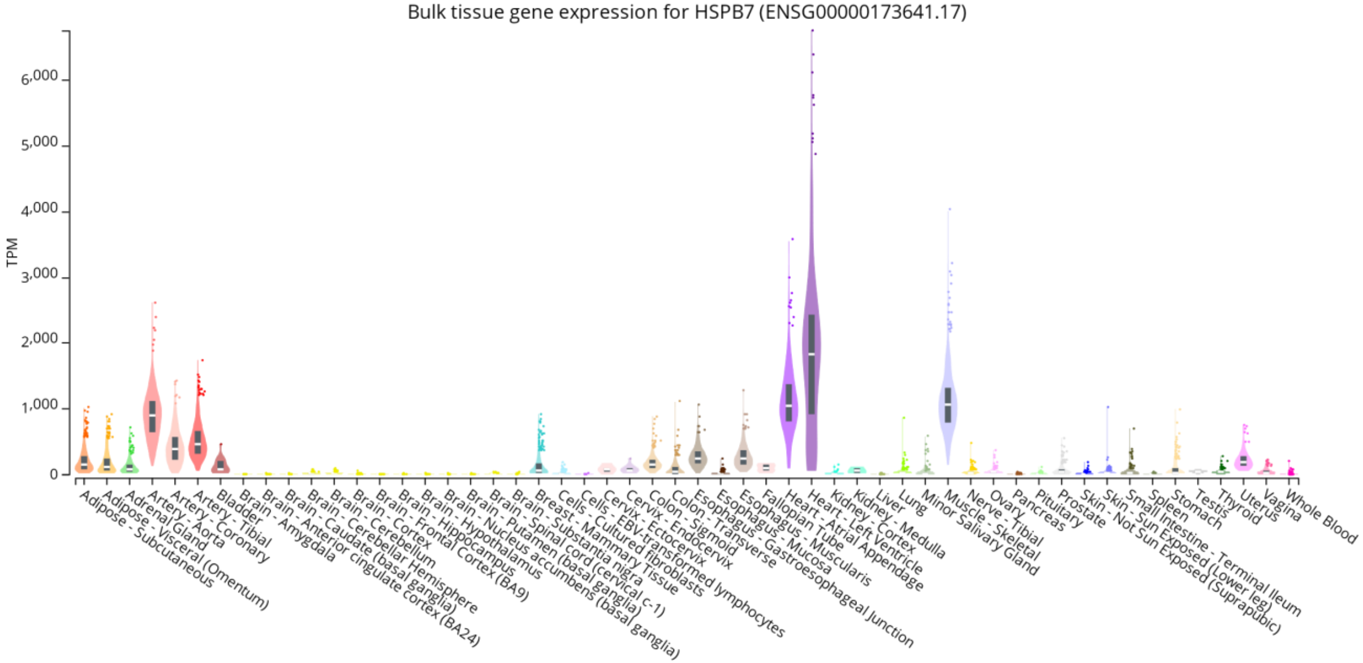

ii) HSPB7 - Enriched in Cardiomyocytes

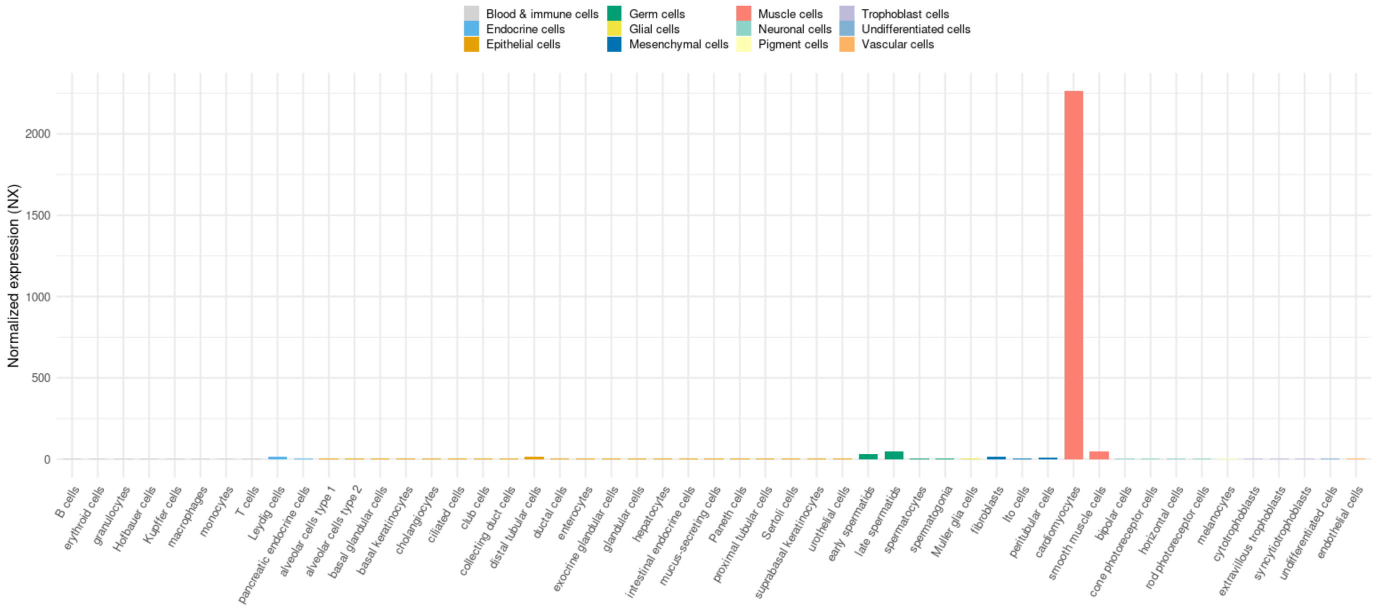

iii) HSPB7 - Enriched in Cardiomyocytes

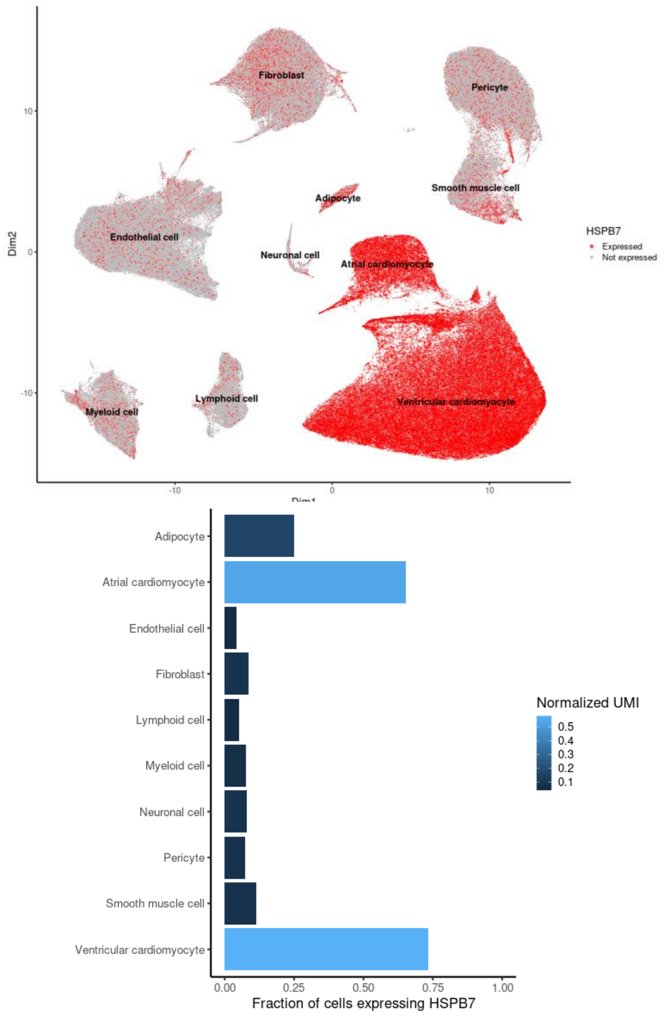

Fig. S4C. Transcriptomics data analysis on *HSPB7*. Data shown are the tissue and single cell type mRNA expression levels based on RNA sequencing data sets from the **i)** GTExPortal (https://gtexportal.org/home/), **ii)** the Human Protein Atlas (https://www.proteinatlas.org/) and **iii)** the Heart Cell Atlas (https://www.heartcellatlas.org/).

i) LMO2 - Ubiquitous. Low tissue specificity

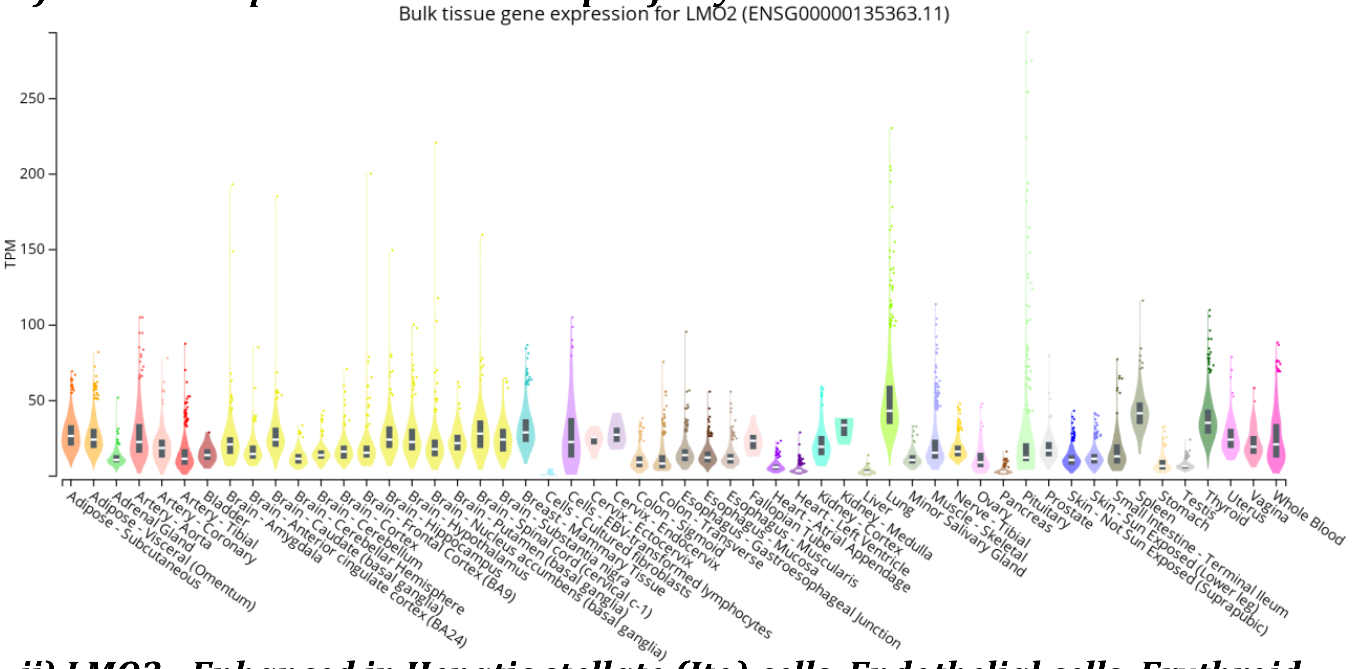

ii) LMO2 - Enhanced in Hepatic stellate (Ito) cells, Endothelial cells, Erythroid cells, Kupffer cells, Microglial cells

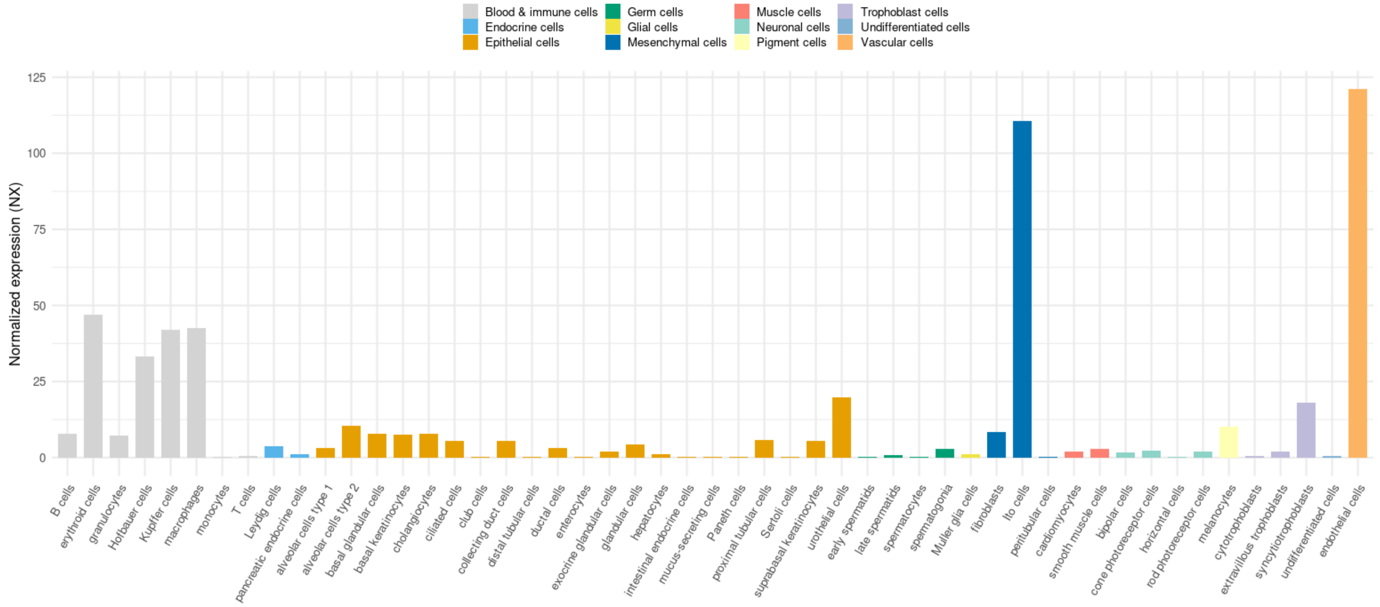

iii) LMO2 – Enriched in Endothelial cells and Myeloid cells

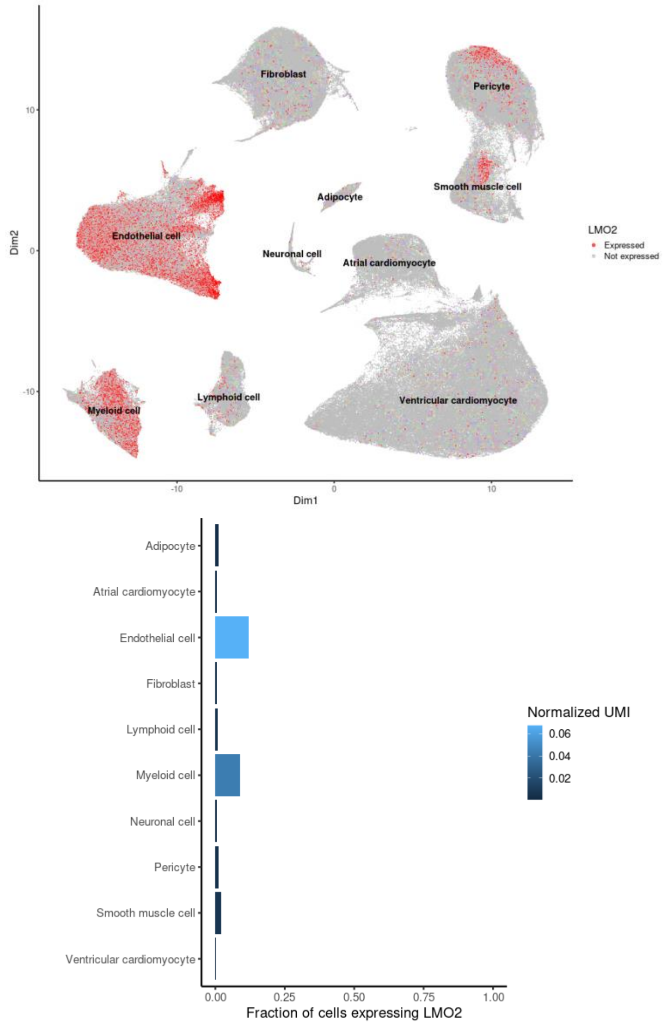

Fig. S4D. Transcriptomics data analysis on *LMO2*. Data shown are the tissue and single cell type mRNA expression levels based on RNA sequencing data sets from the **i)** GTExPortal (https://gtexportal.org/home/), **ii)** the Human Protein Atlas (https://www.proteinatlas.org/) and **iii)** the Heart Cell Atlas (https://www.heartcellatlas.org/).

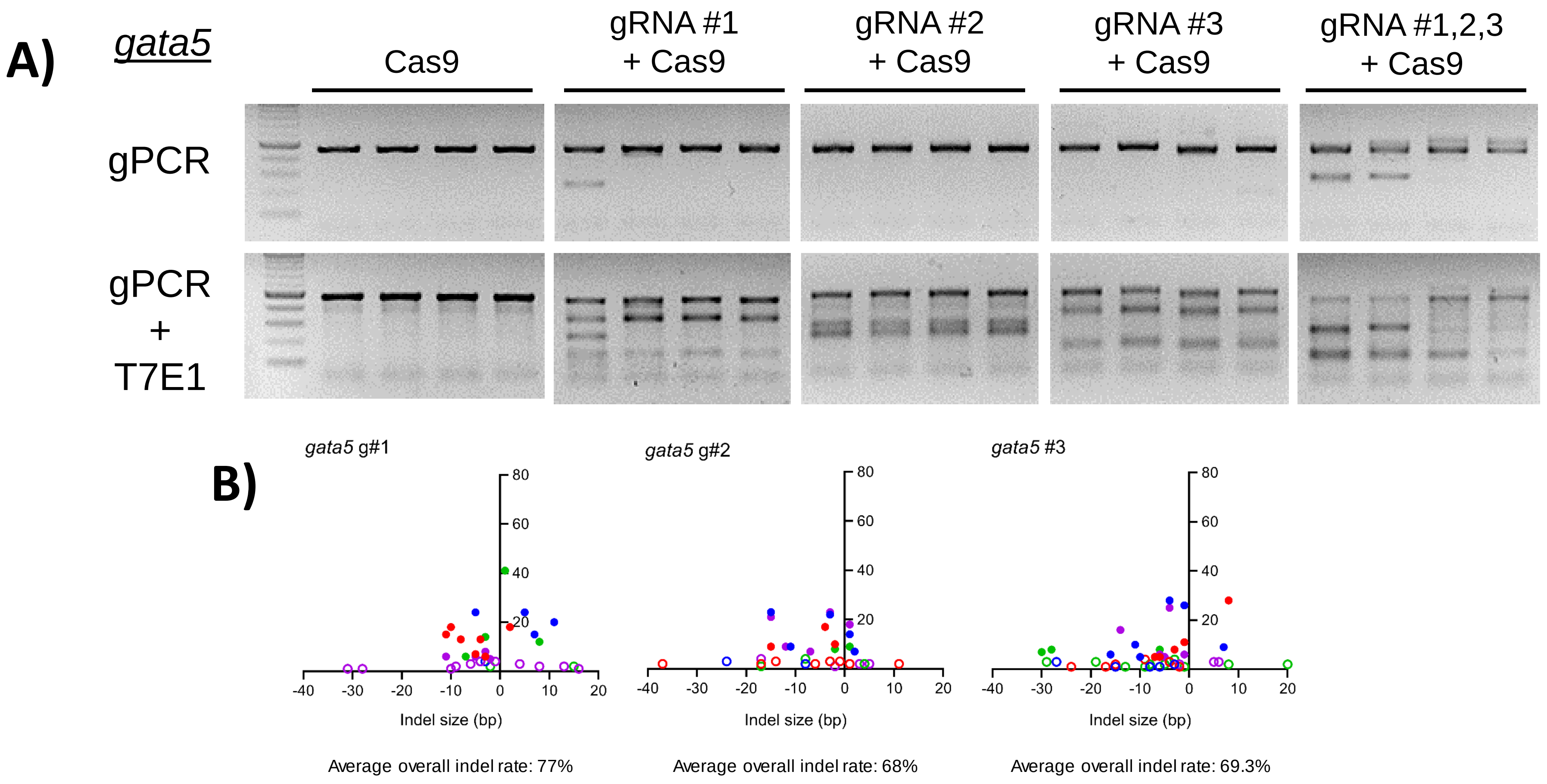

Fig. S5. Measures of mutation efficiency after injection of *gata5* g#1, g#2, and g3# with Cas9 individually or in combination. Panel A) Data shown left to right (top), genomic DNA PCR products from 4 larvae subjected to injection of Cas9 protein alone, *gata5* gRNA #1 + Cas9, *gata5* gRNA #2 + Cas9, *gata5* gRNA #3 + Cas9, and *gata5* gRNA #1,2,3 + Cas9. Note the presence of extra bands, particularly after injection of the *gata5* gRNA #1,2,3 + Cas9 indicating the presence of large deletions in the *gata5* gene. Data shown left to right (bottom), are the same samples after T7 endonuclease I assay resulting in the cleavage of heteroduplex DNA. The presence of multiple bands in all gRNA + Cas9 samples indicates the presence of multiple small deletions in the *gata5* gene, which are not visible after injection of the Cas9 protein alone. Collectively these data that 16/16 (100% across all guide combinations) animals showed mutation in the presence of gRNAs + Cas9, but zero in the Cas9-only control group animals (4/4 or 100%). These data also support the use of this approach to rapidly indicate effective mutagenesis in each of the candidate genes as part of the higher throughput work flow used to support early target validation. **Note:** the data for the Cas9 control, *gata5* gRNA #1 + Cas9 and *gata5* gRNA #1,2,3 + Cas9 are also shown in Fig. 2 of the main manuscript. **Panel B)** Scatter plot graphs in each panel show the indel size and frequency in the PCR products from the three individual gRNAs used for gata5 + Cas9 group (n=4) per gene assessed using ICE. Data points with same colour indicate the indels identified in the same individual embryo within the group. The size ranges of deletions between gRNA target sites are shaded. Indels with less than 5% frequency are presented by open circles.

A)

	Malformations at 2 days post fertilisation (2dpf)											
	n-number (scored)	% Lethality (by 2dpf)	% Larvae Abnormal	Body Shape	Somites	Notochord	Tail	Heart	Face	Neural Tube	Arches/Jaws	Abdomen distended/yolk oedema
Cas9	110	0%	3%	0%	2%	2%	0%	3%	2%	2%	1%	1%
gata5 g#1 + Cas9	95	0%	68%	0%	0%	0%	0%	68%	0%	0%	0%	65%
gata5 g#2 + Cas9	57	0%	18%	0%	0%	0%	0%	18%	0%	0%	0%	7%
gata5 g#3 + Cas9	60	0%	18%	0%	0%	0%	0%	18%	0%	0%	0%	17%
gata5 g#1,2,3 +Cas9	102	2%	87%	0%	3%	1%	0%	87%	3%	1%	0%	94%

Aii

	Heart-specific malformations at 2dpf						
	n-number (scored)	% Abnormal	Slow Heartbeat	No Heartbeat	Pericardial Sac-Swollen	Chambers not well defined	Misshapen
Cas9	110	3%	0%	0%	3%	1%	2%
gata5 g#1 + Cas9	97	54%	3%	10%	31%	2%	37%
gata5 g#2 + Cas9	57	18%	0%	0%	18%	0%	0%
gata5 g#3 + Cas9	60	18%	0%	0%	17%	0%	3%
gata5 g#1,2,3 +Cas9	100	87%	19%	8%	73%	10%	53%

Aiii

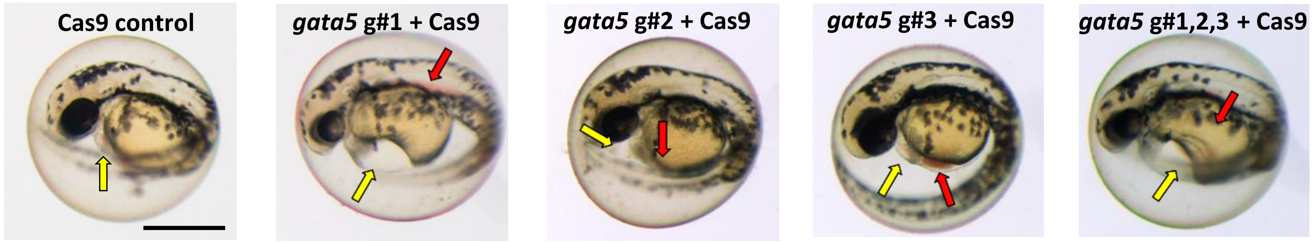

B)

	Malformations at 2 days post fertilisation (2dpf)											
	n-number (scored)	% Lethality (by 2dpf)	% Larvae Abnormal	Body Shape	Somites	Notochord	Tail	Heart	Face	Neural Tube	Arches/Jaws	Abdomen distended/yolk oedema
Cas9	42	2%	0%	0%	0%	0%	0%	0%	0%	0%	0%	0%
api5 g#1 + Cas9	44	0%	0%	0%	0%	0%	0%	0%	0%	0%	0%	0%
api5 g#2 + Cas9	53	19%	0%	0%	0%	0%	0%	0%	0%	0%	0%	0%
api5 g#3 + Cas9	44	2%	2%	0%	2%	2%	0%	2%	2%	2%	2%	2%
api5 g#1,2,3 + Cas9	50	14%	0%	0%	0%	0%	0%	0%	0%	0%	0%	0%

Bii

	Heart-specific malformations at 2dpf						
	n-number (scored)	% Abnormal	Slow Heartbeat	No Heartbeat	Pericardial Sac-Swollen	Chambers not well defined	Misshapen
Cas9	44	0%	0%	0%	0%	0%	0%
api5 g#1 + Cas9	43	0%	0%	0%	0%	0%	0%
api5 g#2 + Cas9	43	0%	0%	0%	0%	0%	0%
api5 g#3 + Cas9	43	2%	0%	2%	0%	2%	2%
api5 g#1,2,3 + Cas9	41	0%	0%	0%	0%	0%	0%

Biii

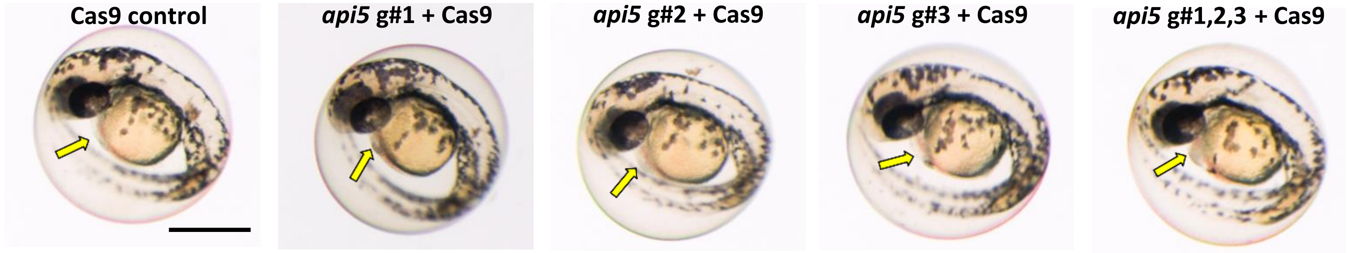

Fig. S6A and B. Comparison of morphological endpoints measured in 2dpf zebrafish. Panel A) General whole body morphological endpoints measured following injection of Cas9 alone, or injection of *gata5* gRNAs 1, 2 and 3 in isolation or combined (g#1, g#2, g#3 or g#1,2,3 respectively) all with Cas9. Data are shown as the % incidence of abnormalities under each of the morphological categories, with shades from white (0%) through to red (100%) providing an indication of the proportion of animals exhibiting a malformation within that category. **Ai**: Expansion of the heart-specific endpoints measured showing the full range of endpoints scored within this category, across the same treatments. **Aii**: Example images of typical 2dpf animals within each treatment category. The yellow arrows show the position of the pericardial membrane and the extent of pericardial oedema in that animal, and the red arrows shows regions of blood pooling; **Panel B)** As Panel A, but for *api5*. More detailed whole body morphological endpoint scoring and cardiovascular functional assessment was undertaken on the two guide combinations showing the strongest phenotypes from the 2dpf data. Scale bar shown in the first image of each panel represents 500µm.

C)

Malformations at 2 days post fertilisation (2dpf)	n-number (scored)	% Lethality (by 2dpf)	% Larvae Abnormal	Body Shape	Somites	Notochord	Tail	Heart	Face	Neural Tube	Arches/Jaws	Abdomen distended/yolk oedema
Cas9	48	0%	0%	0%	0%	0%	0%	0%	0%	0%	0%	0%
hspb7 g#1 + Cas9	47	0%	2%	0%	0%	0%	0%	2%	0%	0%	0%	0%
hspb7 g#2 + Cas9	48	0%	2%	2%	2%	2%	2%	2%	2%	2%	2%	0%
hspb7 g#3 + Cas9	41	0%	12%	0%	10%	7%	5%	10%	10%	10%	10%	10%
hspb7 g#1,2,3 + Cas9	48	0%	17%	10%	13%	10%	13%	10%	13%	13%	8%	13%

Cii

Heart-specific malformations at 2dpf	n-number (scored)	% Abnormal	Slow Heartbeat	No Heartbeat	Pericardial Sac-Swollen	Chambers not well defined	Misshapen
Cas9	48	0%	0%	0%	0%	0%	0%
hspb7 g#1 + Cas9	47	2%	0%	0%	2%	0%	0%
hspb7 g#2 + Cas9	48	2%	0%	2%	0%	2%	2%
hspb7 g#3 + Cas9	41	10%	5%	0%	5%	0%	5%
hspb7 g#1,2,3 + Cas9	48	10%	0%	4%	10%	6%	8%

Ciii

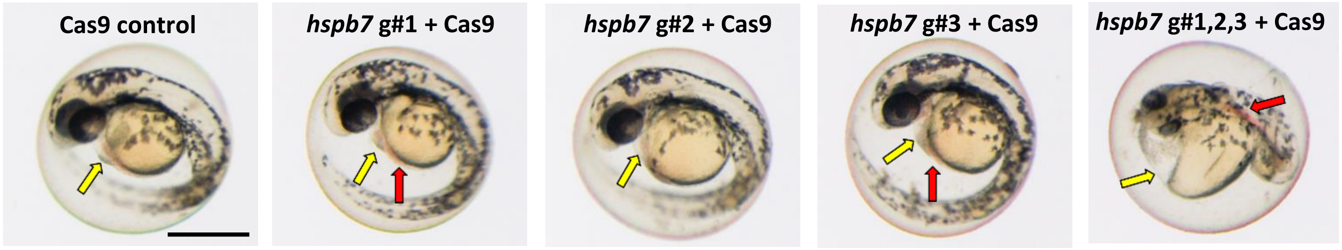

D)

Malformations at 2 days post fertilisation (2dpf)	n-number (scored)	% Lethality (by 2dpf)	% Larvae Abnormal	Body Shape	Somites	Notochord	Tail	Heart	Face	Neural Tube	Arches/Jaws	Abdomen distended/yolk oedema
Cas9	46	8%	7%	0%	2%	2%	2%	7%	2%	2%	2%	2%
lmo2 g#1 + Cas9	3	94%	33%	0%	33%	33%	33%	33%	33%	33%	33%	33%
lmo2 g#2 + Cas9	45	10%	91%	2%	20%	7%	11%	91%	24%	11%	9%	18%
lmo2 g#3 + Cas9	43	24%	91%	5%	12%	5%	5%	91%	14%	9%	7%	44%
lmo2 g#1,2,3 + Cas9	12	76%	92%	25%	67%	50%	58%	92%	58%	58%	42%	58%

Dii

Heart-specific malformations at 2dpf	n-number (scored)	% Abnormal	Slow Heartbeat	No Heartbeat	Pericardial Sac-Swollen	Chambers not well defined	Misshapen
Cas9	46	7%	2%	0%	7%	0%	4%
lmo2 g#1 + Cas9	3	33%	0%	33%	33%	33%	33%
lmo2 g#2 + Cas9	45	91%	7%	7%	73%	11%	82%
lmo2 g#3 + Cas9	43	91%	2%	2%	60%	2%	81%
lmo2 g#1,2,3 + Cas9	12	92%	8%	17%	58%	25%	92%

Diii

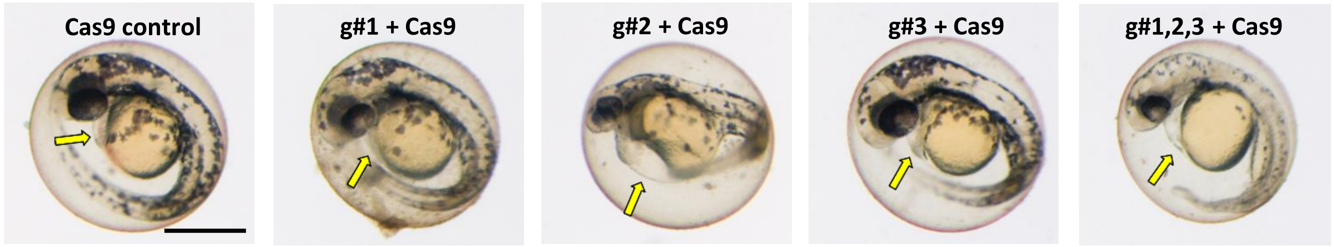

Fig. S6C and D. Comparison of morphological endpoints measured in 2dpf animals. Panel C) General whole body morphological endpoints measured following injection of Cas9 alone, or injection of *hspb7* gRNAs 1, 2 and 3 in isolation or combined (g#1, g#2, g#3 or g#1,2,3 respectively) all with Cas9. Data are shown as the % incidence of abnormalities under each of the morphological categories, with shades from white (0%) through to red (100%) providing an indication of the proportion of animals exhibiting a malformation within that category. **Ci**: Expansion of the heart-specific endpoints measured showing the full range of endpoints scored within this category, across the same treatments. **Cii**: Example images of typical 2dpf animals within each treatment category. The yellow arrows show the position of the pericardial membrane and the extent of pericardial oedema in that animal, and the red arrows shows regions of blood pooling; **Panel D)** as Panel C) but for *lmo2*. More detailed whole body morphological endpoint scoring and cardiovascular functional assessment was undertaken on the two guide combinations showing the strongest phenotypes from the 2dpf data. Scale bar shown in the first image of each panel represents 500µm.

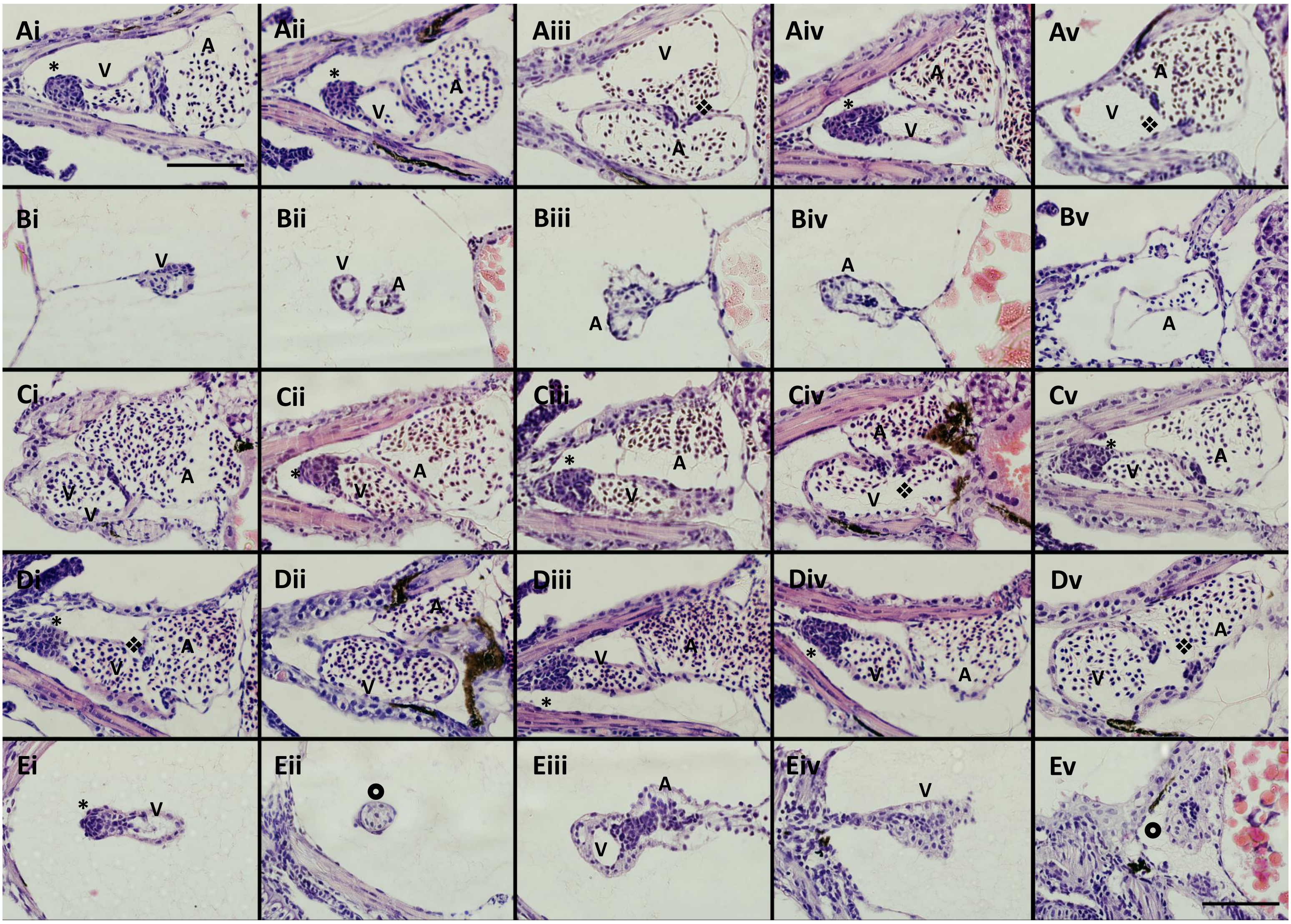

Fig. S7. Haematoxylin and eosin stained coronal sections through the hearts of 4dpf CRISPR-mutant zebrafish. In each row are sections from 5 animals (i-v) subjected to A) Cas9 injection; B) gata5 g#1,2,3 + Cas9 injection; C) *api5* g#1,2,3 + Cas9 injection; D) *hspb7* g#1,2,3 + Cas9 injection and E) *lmo2* g#1,2,3 + Cas9 injection. In each panel, animals are orientated with the head to the left, and viewed in the dorsal plane at a magnification of 40x (Scale bar shown in top left and bottom right images represents 200µm). On various panels, labelled structures include the ventricle (v); atrium (A); bulbus arteriosus (*); atrioventricular value (❖); and unspecified heart tissue (⦿).

Cas9

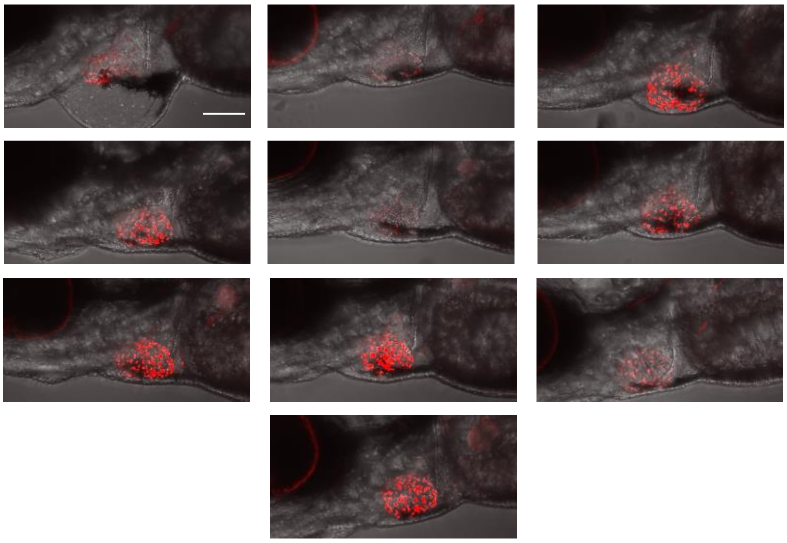

gata5

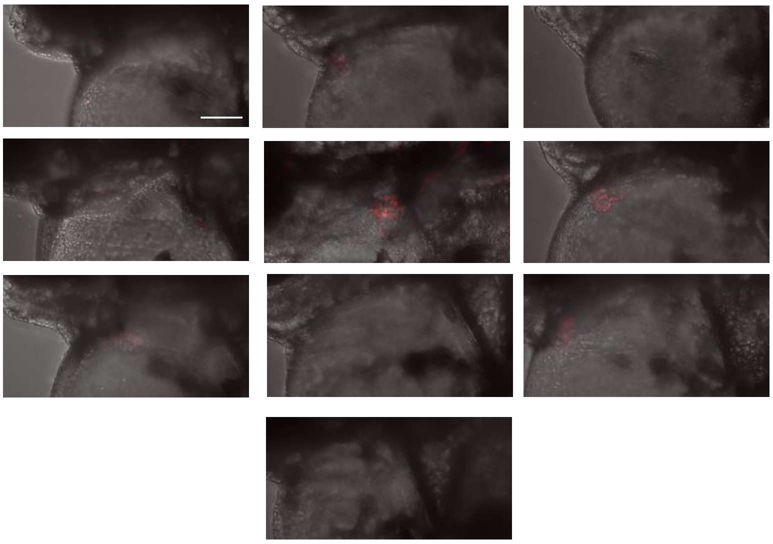

api5

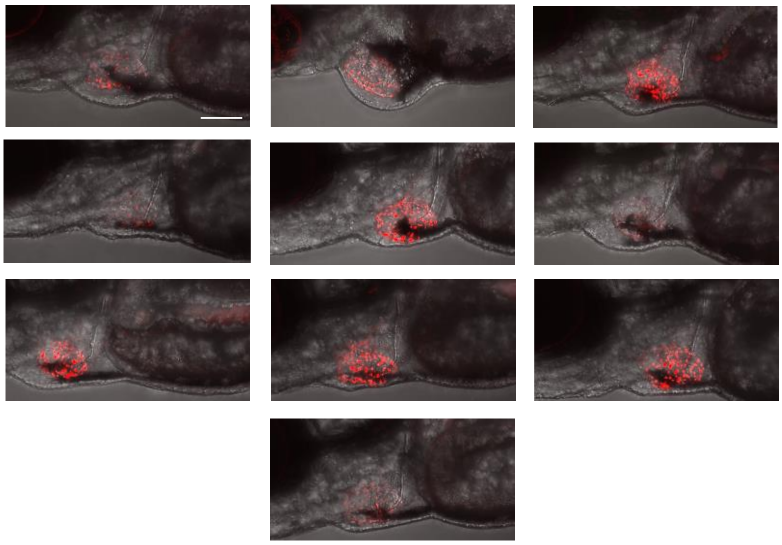

Fig. S8A. Confocal maximum intensity projection images of hearts from *cmlc2::DsRed2-nuc* mutant larvae. The images shown are the combined transmitted light and *cmlc2::DsRed2-nuc* fluorescence signals from 10 randomly sampled larvae per treatment, with cardiomyocytes fluorescing in red, especially prominently in the ventricle (all laser settings identical). The top set of images shows hearts from Cas9 injected (injection controls) and then from embryos injected with *gata5* g#1,2,3 + Cas9 (middle), and *api5* g#1,2,3 + Cas9, (bottom). Scale bar shown in the first image of each panel represents 100 µm.

Cas9

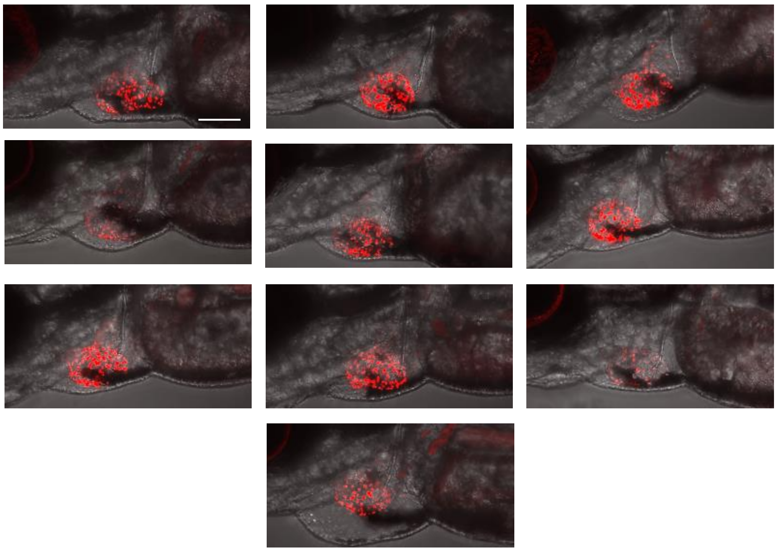

hspb7

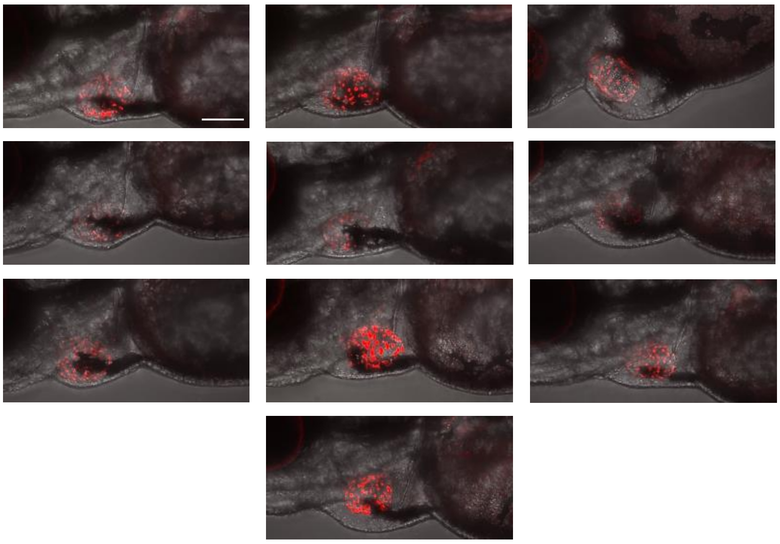

lmo2

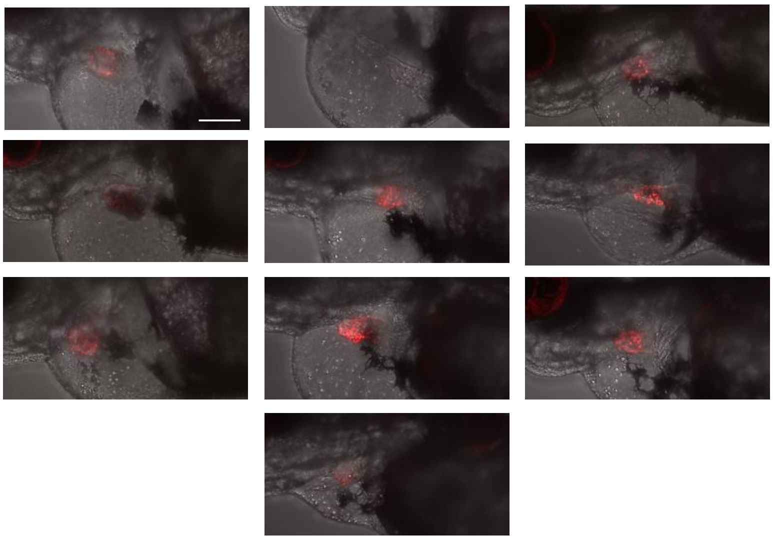

Fig. S8B. Confocal maximum intensity projection images of hearts from *cmlc2::DsRed2-nuc* mutant larvae. The images shown are the combined transmitted light and *cmlc2::DsRed2-nuc* fluorescence signals from 10 randomly sampled larvae per treatment, with cardiomyocytes fluorescing in red, especially prominently in the ventricle (all laser settings identical). The top set of images shows hearts from Cas9 injected (injection controls) and then from embryos injected with *hspb7* g#1,2,3 + Cas9 (middle) and *lmo2* g#2 + Cas9 (bottom). Scale bar shown in the first image of each panel represents 100 µm.

SUPPLEMENTARY VIDEOS

Video 1) V1 Cas9 heart beat (control)

Video 2) V2 Cas9 blood flow (control)

Video 3) V3 gata5 g#1 heart beat

Video 4) V4 gata5 g#1 blood flow

Video 5) V5 gata5 g#123 heart beat

Video 6) V6 gata5 g#123 blood flow

Video 7) V7 api5 g#1 heart beat

Video 8) V8 api5 g#1 blood flow

Video 9) V9 api5 g#123 heart beat

Video 10) V10 api5 g#123 blood flow

Video 11) V11 hspb7 g#3 heart beat

Video 12) V12 hspb7 g#3 blood flow

Video 13) V13 hspb7 g#123 heart beat

Video 14) V14 hspb7 g#123 blood flow

Video 15) V15 lmo2 g#2 heart beat

Video 16) V16 lmo2 g#2 blood flow

Video 17) V17 lmo2 g#123 heart beat

Video 18) V18 lmo2 g#123 blood flow

SUPPLEMENTARY DATASETS

Data S1) Data S1_Network Analysis.xlsx

Data S2) Data S2_Differential Expression.xlsx

Data S3) Data S3_Genetic Associations.xlsx

Data S4) Data S4_Homologies.xlsx
